# A review on the role of long non-coding RNA and microRNA network in clear cell renal cell carcinoma and its tumor microenvironment

**DOI:** 10.1186/s12935-023-02861-6

**Published:** 2023-02-02

**Authors:** Qi Zhang, Hao Ren, Luqi Ge, Wen Zhang, Feifeng Song, Ping Huang

**Affiliations:** 1 Department of Pharmacology, College of Pharmaceutical Sciences, Zhejiang University of Technology, Hangzhou, China; 2 Center for Clinical Pharmacy, Cancer Center, Department of Pharmacy, Zhejiang Provincial People’s Hospital, Affiliated People’s Hospital, Hangzhou Medical College, Hangzhou, China; 3 Key Laboratory of Endocrine Gland Diseases of Zhejiang Province, Hangzhou, China

**Keywords:** Clear cell renal cell carcinoma, lncRNA, miRNA, Regulatory network, TME

## Abstract

Renal cell carcinoma (RCC) is the second lethal urogenital malignancy with the increasing incidence and mortality in the world. Clear cell renal cell carcinoma (ccRCC) is one major subtype of RCC, which accounts for about 70 to 80% of all RCC cases. Although many innovative therapeutic options have emerged during the last few decades, the efficacy of these treatments for ccRCC patients is very limited. To date, the prognosis of patients with advanced or metastatic ccRCC is still poor. The 5-year survival rate of these patients remains less than 10%, which mainly attributes to the complexity and heterogeneity of the tumor microenvironment (TME). It has been demonstrated that long non-coding RNAs (lncRNAs) perform an indispensable role in the initiation and progression of various tumors. They mostly function as sponges for microRNAs (miRNAs) to regulate the expression of target genes, finally influence the growth, metastasis, apoptosis, drug resistance and TME of tumor cells. However, the role of lncRNA/miRNA/mRNA axis in the TME of ccRCC remains poorly understood. In this review, we summarized the biological function of lncRNA/miRNA/mRNA axis in the pathogenesis of ccRCC, then discussed how lncRNA/miRNA/mRNA axis regulate the TME, finally highlighted their potential application as novel biomarkers and therapeutic targets for ccRCC.

## Introduction

Renal cell carcinoma (RCC) is one of the most lethal urogenital malignancies with an increasing morbidity and mortality in the world [1]. According to the global cancer statistics report, RCC is the ninth most frequently diagnosed cancer in men, which has approximately 432,000 new cases and 180,000 deaths in 2020 [2]. The major histological subtype of RCC is clear cell RCC (ccRCC), which accounts for about 75% of all RCC cases, followed by papillary RCC (pRCC) and chromophobe RCC (chRCC) (representing ~ 15–20% and ~ 5% of RCC, respectively) [3]. Due to the high rate of recurrence or metastasis and difficulties in the early diagnosis, it is conceivable that the prognosis of patients with ccRCC is still very poor. Until now, radical nephrectomy remains the best treatment and crucial intervention for local ccRCC. However, up to one-third of patients are initially diagnosed with metastatic ccRCC, at which point they are no longer suitable for surgical treatment [4, 5]. Moreover, roughly 20 to 30% of patients will relapse within 2 years after radical nephrectomy, and almost all of them are extremely resistant to both chemotherapeutics and radiation therapy [4, 6]. During the last few decades, owing to the rapid development of targeted and immunological drugs, the therapeutic options for ccRCC patients have been greatly expanded. Unfortunately, the efficacy of these treatments for patients with advanced or metastatic ccRCC is still very limited, which mainly attributes to the complexity and heterogeneity of tumor microenvironment (TME). Therefore, it is extremely urgent to understand the molecular mechanism in the tumorigenesis and progression of ccRCC, especially its dynamic TME, which may be helpful to discover novel effective biomarkers and therapeutic targets of ccRCC.

The carcinogenesis of ccRCC is a complicated biological process, which is closely associated with gene mutation, genome instability, and epigenetic disorder [7]. Some important genes are well known to participate in the occurrence and development of ccRCC, for instance, the von Hippel-Lindau (*VHL*) gene. It has been demonstrated that *VHL* is mutated in ccRCC, and its mutation leads to an activation of hypoxia-inducible factor (HIF), thereby inducing the expression alteration of angiogenic factors including vascular endothelial growth factor (VEGF) and platelet-derived growth factor (PDGF) [8]. Moreover, *VHL* mutation also activates the mammalian target of rapamycin (mTOR) signaling pathway, which in turn upregulates HIF and angiogenesis, subsequently accelerating the progression of ccRCC [9]. Consequently, drugs directly target VEGF/VEGF receptor (VEGFR), PDGF/PDGF receptor (PDGFR), and the mTOR pathway have been dramatically developed [9]. Nevertheless, there are no credible diagnostic and prognostic biomarkers for ccRCC that have yet been applied into clinical practice. In recent years, the epigenetics of ccRCC, including non-coding RNAs (ncRNAs), have greatly attracted the attention of researchers. A large amount of ncRNAs are found to be aberrantly expressed in diverse tumors, indicating ncRNAs play critical roles in tumorigenesis and development [10–12]. Thus, further identifying and understanding the function of ncRNAs may contribute to the early diagnosis and treatment of ccRCC.

NcRNAs can be classified into long ncRNAs (lncRNAs) and small ncRNAs (sncRNAs) based on their length [13]. As for lncRNAs, there are more than 200 nucleotides in their molecular sequences, but they are not responsible for protein-coding [14]. LncRNAs have been found to be localized both in the nuclear and cytoplasmic compartments. Studies have demonstrated that lncRNAs can influence many cellular processes depending on their location. In the nucleus, lncRNAs can regulate gene expression by recruiting chromatin-modifying complexes, or by changing the spatial conformation of chromosomes [15]. Moreover, they can also influence the efficiency of transcriptional factors or pre-mRNA spliceosomes to modulate mRNA expression. In the cytoplasm, lncRNAs can regulate transcription by influencing mRNA stability, mRNA translation, or miRNA binding [14]. Additionally, few lncRNAs can also be translated into biological active small peptides [16]. In contrast to lncRNAs, sncRNAs are a series of RNAs with about 19 to 25 nucleotides in length. MicroRNAs (miRNAs) are one subclass of sncRNAs, which are small single-stranded RNA molecules and exert their functions exclusively at the post-transcriptional level [13]. MiRNAs can induce the transcriptional silence of target genes by binding to their 3’untranslated region (3’UTR) sequences [17]. More importantly, a growing body of studies have discovered that miRNAs have a close interaction with lncRNAs. LncRNAs can act as sponges to interact with miRNAs, and also have an effect on their production and degradation, eventually leading to the alteration of target genes [18]. In addition, both lncRNAs and miRNAs are known to be involved in cell proliferation, apoptosis, metastasis, and tumor microenvironment (TME) [19, 20]. Therefore, regulation of aberrant lncRNAs and miRNAs may alleviate the initiation and development of various tumors. Although the current knowledge of lncRNAs and miRNAs is still inadequate, an increasing number of studies have emphasized their indispensable role in tumorigenesis. In this review, we aim to summarize the latest advances regarding lncRNA/miRNA/mRNA axis in the pathogenesis of ccRCC, then discuss how lncRNA/miRNA/mRNA axis regulate TME, and finally highlight their potential application as novel biomarkers and therapeutic targets for ccRCC.

### The regulatory mode between lncRNA and miRNA

**Fig. 1:**
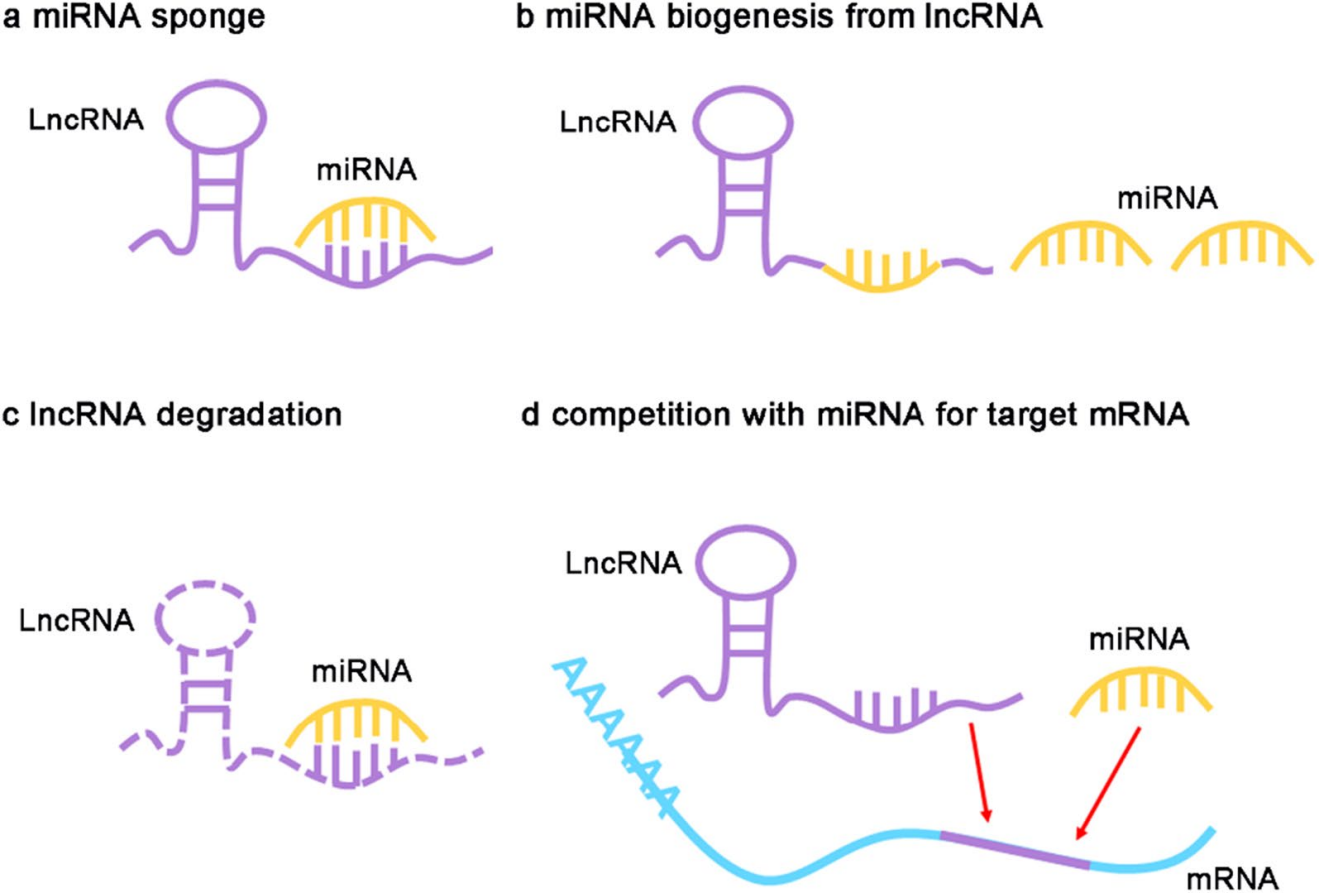
The regulatory mode between lncRNA and miRNA. **a** LncRNA acts as a sponge for miRNA, which prohibits the binding between miRNA and its targets. **b** LncRNA can be digested to produce miRNA and influence the transcriptional expression of downstream genes. **c** MiRNA can induce the degradation of lncRNA via binding to lncRNAs, and eventually suppress the mRNA levels of target genes. **d** LncRNA can competitively interact with the target genes of miRNA

Based on the previous reports, the interaction between lncRNA and miRNA can be summarized as four regulatory modes, namely sponges for miRNA, production of miRNA, degradation of lncRNA, and competition with miRNA for target genes (Fig. 1). To be specific, lncRNAs can act as miRNA sponges, which directly target miRNAs to suppress the combination between miRNAs and their target genes, subsequently leading to the expression changes of target genes. Up to now, most studies have been predominantly focused on this regulatory mode, which can be defined as lncRNA/miRNA/mRNA axis [21, 22]. In addition to serving as miRNA sponges, lncRNAs are also responsible for the biogenesis of miRNAs because some nucleotides in the lncRNA sequences were the same as miRNAs. In this process, lncRNAs can be cleaved into miRNAs by Dicer and/or Drosha, then alter the transcriptional expression of downstream genes [23]. In contrast, miRNAs also can induce the degradation of lncRNAs through binding with lncRNAs, and eventually repress the mRNA expression of downstream genes [24]. Additionally, lncRNAs can directly interact with the target genes of miRNAs, which restrain the binding between miRNAs and their target genes, and finally affect the activity of miRNAs [25, 26].

### Biological role of lncRNA/miRNA/mRNA axis in ccRCC

LncRNA/miRNA/mRNA axis plays a vital role in the initiation and development of different carcinomas, such as ccRCC. Studies have shown that some oncogenic miRNAs are upregulated in ccRCC, while some tumor suppressor miRNAs are downregulated. In that case, lncRNAs combine with oncogenic miRNAs will result in tumor inhibition, and those interact with suppressor miRNAs will influence the tumor in reverse.

### Role in promoting tumor progression

**Fig. 2:**
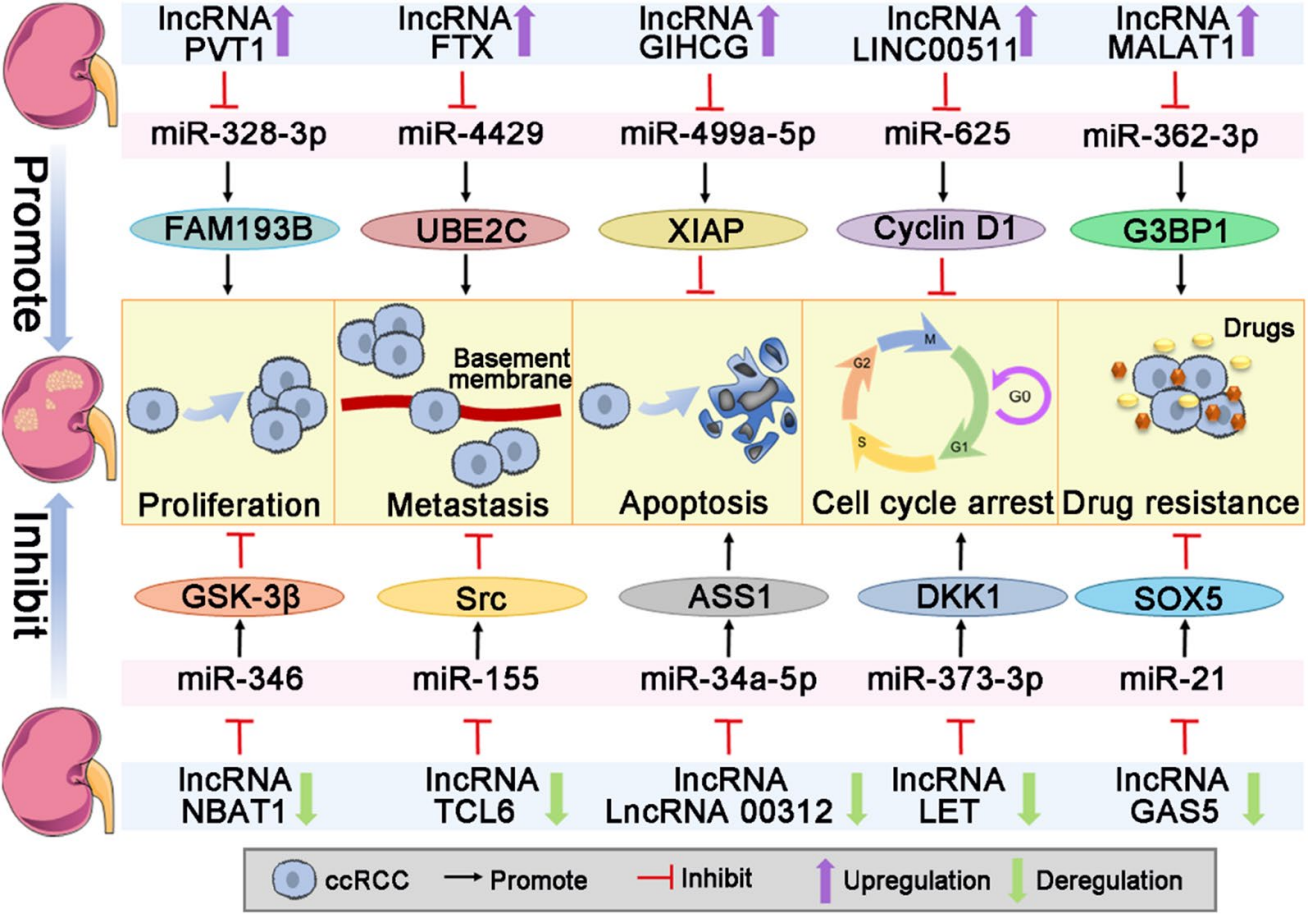
Role of lncRNA/miRNA/mRNA axis in the progression of ccRCC. LncRNA/miRNA/mRNA axis plays an important role in promoting or inhibiting the progression of ccRCC, which has an effect on cell proliferation, metastasis, apoptosis, cell cycle and drug resistance

Previous studies have demonstrated that lncRNA/miRNA/mRNA axis is critical in the progression of ccRCC, which can facilitate cell proliferation, migration, invasion, cell cycle and inhibit apoptosis (Fig. 2). Xie et al. have illustrated that lncRNA PVT1 could promote ccRCC cell proliferation by directly targeting miR-328-3p, then induce the expression of family with sequence similarity 193 member B (FAM193B), and activate the PI3K/AKT and MAPK/ERK signal pathways [27]. PI3K/AKT and MAPK/ERK pathways are crucial for cell proliferation, metastasis, and angiogenesis, and they have been found to be dysregulated in ccRCC [28]. On the other hand, Chen et al. have discovered that PVT1 was remarkably upregulated in ccRCC tissues based on the high-throughput analysis, and its upregulation was closely associated with the lower overall survival (OS) rate of ccRCC patients [29]. Furthermore, PVT1 also functioned as a competing endogenous RNA (ceRNA) of miR-145-5p to increase the expression of T-box transcription factor 15 (TBX15) in ccRCC cells [30]. TBX15 expression is positively related to the poor prognosis of ccRCC patients, who have a shorter OS and disease-free survival (DFS) [30]. Overexpression of PVT1 could promote the migration and invasion of ccRCC cells via inhibiting miR-16-5p [31].

LncRNA MALAT1 expression is markedly increased in ccRCC tissues than that in the normal controls, and its high expression promotes ccRCC cell proliferation and invasion by sponging miR-200s, thereby inducing the expression of zinc finger E-box-binding homeobox 2 (ZEB2) [10]. ZEB2 acts as a DNA-binding transcriptional repressor, which is elevated in ccRCC and negatively correlated with the tumor metastasis and prognosis [32]. Chen et al. have reported that ZEB2 could be regulated by miR-30a-5p and miR-206, which influenced the proliferation, migration and invasion of ccRCC cells [33, 34]. Notably, the high level of MALAT1 is also associated with the worse prognosis of ccRCC patients [35]. Besides, MALAT1 could competitively sponge miR-194-5p to activate activin A receptor type 2B (ACVR2B), which resulted in the increase of cell proliferation and suppression of apoptosis [36]. The promoting effect of ACVR2B on ccRCC progression is dependent on the activation of activin A, which can be activated by the dimerization of activin receptor types I and II [36]. Additionally, silencing of MALAT1 in ccRCC cells could enhance the sensitivity to sunitinib by modulating miR-362-3p/Ras-GTPase-activating SH3-domain-binding protein 1 (G3BP1) axis [37]. G3BP1 is important for the progression and metastasis of ccRCC, which can facilitate cell proliferation, migration and invasion by regulating IL-6/G3BP1/STAT3 signaling pathway [38]. Furthermore, lncRNA DARS-AS1 can upregulate the level of aspartyl-tRNA synthetase (DARS) through sequestering miR-194-5p, then contribute to the malignant progression of ccRCC [39]. Zhu et al. have found that lncRNA GIHCG could sponge miR-499a-5p to boost the expression of X-linked inhibitor of apoptosis protein (XIAP), thereby promoting the proliferation, migration and cell cycle of ccRCC cells and inhibiting apoptosis [40]. In addition, high expression of lncRNA LINC00511 could accelerate ccRCC cell proliferation and inhibit cell cycle arrest at G0-G1 by modulating miR-625/Cyclin D1 signaling [41]. A recent study demonstrated that lncRNA FTX is abnormally upregulated in ccRCC, which promotes the viability, migration and invasion of ccRCC by sponging miR-4429 to induce the expression of ubiquitin-mediated proteolysis genes (UBE2C) [42]. To conclude, numerous lncRNAs have a positive effect on the progression of ccRCC through regulating different miRNA/mRNA axis (Table 1).

**Table 1:** Role of lncRNA/miRNA/mRNA axis in promoting ccRCC progression

LncRNA	MiRNA	Gene	Biological behavior of ccRCC	Types of ccRCC tissues and cell lines	Refs.
PVT1	miR-328-3p, miR-145-5p, miR-16-5p, miR-200s	FAM193B, TBX15, BMI1, ZEB1, ZEB2	Proliferation, migration, invasion, apoptosis, cell cycle, EMT^a^	Tissues: 210 NT^b^ Cells: 769-P, UMRC6, RCCJF, A498, ACHN, 786-O, OS-RC-2, Caki-1, HEK-293T, HK-2	[27, 30, 31, 43]
HILAR	miR-613, miR-206, miR-1-1-3p	Jagged1, CXCR4	Proliferation, migration, invasion, EMT	Cells: ACHN, Caki-1, SN12-PM6	[44]
TUG1	miR-299-3p, miR-9, miR-31-5p, miR-196a	VEGFR, YAP, FLOT1, AKT, ERK, JNK	Proliferation, migration, invasion, apoptosis, autophagy, EMT	Tissues: 99 NT Cells: ACHN, OS-RC-2, 786-O, 769-P, A498, A704, HEK-293, HK-2	[45–48]
ITGB2-AS1	miR-328-5p	HMGA1	Proliferation, apoptosis	Tissues: 33 NT Cells: OS-RC-2, SW839, A498, SN12-PM6, Caki-1, HK-2	[49]
LUCAT1	miR-495-3p, miR-375	SATB1, YAP1	Proliferation, migration, invasion	Tissues: 126 NT Cells: 786-O, Caki-1, A498, 769-P, ACHN, HK-2	[50, 51]
MEG8	miR-495-3p	G3BP1	Proliferation, migration, invasion	Tissues: 62 NT Cells: A498, Caki-1,786-O, 769-P, ACHN	[52]
RP11-436H11.5	miR-335-5p	BCL-W	Proliferation, invasion	Tissues: 20 NT Cells: A498, 786-O, OS-RC-2	[53]
PCED1B-AS1	miR-484	ZEB1	Proliferation, migration, EMT	Tissues: 40 NT Cells: 786-O, A498, ACHN, Caki-1, HK-2	[54]
CRNDE	miR-136-5p	N-Cadherin, Vimentin	Proliferation, migration, invasion, EMT	Tissues: 45 NT Cells: A498, ACHN	[55]
DARS-AS1	miR-194-5p	DARS	Proliferation, apoptosis	Tissues: 70 NT Cells: ClearCa-1, HH332, Caki-1, KMRC-2, KN-41, HK-2	[39]
LINC01094	miR-224-5p, miR-184	CHSY1, SLC2A3	Proliferation, migration, EMT	Tissues: 56 NT Cells: ACHN, 769-P, Caki-1, 786-O, HK-2	[56, 57]
LINC02747	miR-608	TFE3	Proliferation	Tissues: 70 NT Cells: 786-O, ACHN, Caki-1, Caki-2	[58]
LINC00973	miR-7109-3p	Siglec-15	Immune escape	Tissues: 100 NT Cells: 786-O, 769-P, A704, A498, ACNH, Caki-1, Caki-2, RCC4, HK-2	[59]
LINC01426	miR-423-5p	FOXM1	Proliferation, migration	Cells: A498, ACHN, Caki-1, 786-O, HEK-293T, HK-2	[60]
LINC00511	miR-625	CCND1	Proliferation, migration, invasion, apoptosis, cell cycle	Tissues: 49 NT Cells: A498, 786-O, ACHN, Caki-2, HK-2	[41]
HOXA11‐AS	miR-146b-5p	MMP16	Proliferation, invasion, apoptosis, EMT	Tissues: 52 NT Cells: ACHN, 786-O, A498, OS-RC-2, HK-2	[61]
CDKN2B-AS1	miR-141	Cyclin D1, Cyclin D2	Proliferation, migration, invasion, apoptosis	Cells: RPTEC, ACHN, Caki-1	[62]
DNAJC3-AS1	miR-27a-3p	PRDM14	Proliferation, migration, invasion, apoptosis	Tissues: 30 NT Cells: 769-P, ACHN, Caki-1, 786-O, HK-2	[63]
FGD5-AS1	miR-5590-3p	ERK/AKT	Proliferation, migration, invasion, EMT	Tissues: 28 NT Cells: 786-O, ACHN, SN12-PM6, HK-2	[64]
HCG18	miR-152-3p	RAB14	Proliferation, migration, invasion	Tissues: 32 NT Cells: Caki-1, 786-O, 769-P, ACHN, HK-2	[65]
SNHG12	miR-200c-5p, miR-30a-3p, miR-129-5p	COL11A1, RUNX2, WNT2, IGF-1R, MDM4	Proliferation, invasion, apoptosis, cell cycle	Tissues: 148 NT Cells: OS-RC-2, 786-O, Caki-1, Caki-2, A498, ACHN, 769-P, HK-2	[66–68]
SNHG5	miR-205-5p, miR-363-3p	ZEB1, Twist	Proliferation, migration, invasion, apoptosis, EMT	Tissues: 20 NT Cells: ACHN, 786-O, A498, SN12-PM6, SW13, Caki-1, HK-2	[69, 70]
PCAT1	miR-656, miR-539	YAP	Proliferation, migration, invasion	Tissues: 85 NT Cells: 786-O, Caki-2, 769-P, OS-RC-2, ACHN, HK-2	[71]
H19	miR-29a-3p	E2F1	Proliferation, migration, invasion	Tissues: 30 NT Cell: 786-O	[72]
NNT-AS1	miR-137	Y-box	Proliferation, migration and invasion	Tissues: 40 NT Cells: 786-O, OS-RC-2, A498, Caki-1, HEK-293T	[73]
HCP5	miR-214-3p, miR-140-5p	MAPK1, IGF1R	Proliferation, migration, invasion, apoptosis, cell cycle	Tissues: 142 NT Cells: 786-O, Caki-1, Caki-2, ACHN, A498, 769-P, OS-RC-2, HEK-293T, HK-2	[74, 75]
MIR4435-2HG	miR-513a-5p	KLF6	Proliferation, migration, invasion	Tissues: 40 NT Cells: 786-O, 769-P, Caki-1, Caki-2, ACHN, A498, HK-2, HEK-293T	[76]
PCGEM1	miR-433-3p	FGF2	Proliferation, migration, apoptosis	Tissues: 47 NT Cells: OS-RC-2, ACHN, A498, 786-O	[77]
MALAT1	miR-200s, miR-194-5p, miR‐203, miR-205, miR-429, miR-182-5p, miR-1271-5p	ZEB2, ACVR2B, BIRC5, KIAA1324	Proliferation, migration, invasion, apoptosis, cell cycle	Tissues: 531 NT Cells: 786-O, ACHN, SN12-PM6, OS-RC-2, Caki-2, Caki-1, A498, HK-2	[10, 36, 78–82]
RCAT1	miR-214-5p	E2F2	Proliferation, migration, invasion	Tissues: 52 NT Cells: ACHN, 786-O, 769-P, Caki-1, A498, HK-2	[83]
MSC‐AS1	miR-3924	WNT5A	Proliferation, migration	Tissues: 27NT Cells: 786-O, 769-P, A498, Caki-1, HK-2	[84]
ROR	miR-206	VEGF	Proliferation, migration and invasion	Tissues: 36 NT Cells: Caki-1, Caki-2, HK-2	[85]
ZFAS1	miR-10a	SKA1	Proliferation, migration and invasion	Tissues: 20 NT Cells: 786-O, Caki-1, ACHN, HEK-293T, HK-2	[86]
SNHG3	miR-139-5p, miR-10b-5p	TOP2A; BIRC5	Proliferation, migration, invasion	Tissues: 70 NT Cells: A498, ACHN, Caki-1, 786-O, HK-2	[87, 88]
SNHG17	miR-328-3p	H2AX	Proliferation, migration, invasion, apoptosis, cell cycle	Tissues: 84 NT Cells: 786-O, ACHN, Caki-1, 769-P, HK-2	[89]
UCA1	miR-495, miR-182-5p	EZH2, DLL4	Proliferation, migration, cell cycle	Tissues: 130 NT Cells: 786-O, ACHN, Caki-1, Caki-2, HEK-293T, HK-2	[90, 91]
LOXL1-AS1	miR-589-5p	CBX5	Proliferation, migration	Tissues: 60 NT Cells: 786-O, A498, 769-P, HEK-293	[92]
HIF1A-AS2	miR-130a-5p	ERBB2	Proliferation, migration, invasion, apoptosis, cell cycle	Tissues: 42 NT Cells: ACHN, OSRC-2, 786-O, Caki-1, HK-2	[93]
HOTTIP	miR-615-3p	IGF-2	Proliferation, migration, invasion, apoptosis, cell cycle	Tissues: 57 NT Cells: A498, 786-O, Caki-1, Caki-2, ACHN, HK-2, HEK-293T	[94]
LINC00641	miR-340-5p	c-Myc, CyclinD1, MMP-2	Proliferation and invasion	Tissues: 48 NT Cells: GRC-1, 786-O, SN12-PM6, A498, ACHN, HK-2	[95]
SNHG1	miR-129-3p, miR-137 miR-103a	STAT3, PD-L1 HMGA2	Proliferation, migration, invasion, cell cycle, apoptosis, EMT	Tissues: 60 NT Cells: ACHN, A498, 786-O, Caki-1, HK-2	[96–98]
LINC01133	miR-30b-5p	Rab-3D	Proliferation, migration, invasion	Tissues: 34 NT Cells: ACHN, A498, 786-O, SN12-PM6, HK-2	[99]
HOTAIR	miR-217, miR-138, miR-200c, miR-204, miR-124, miR-203, miR-141	HIF-1α, AXL, EZH2, VIM, ZEB1, ADAM9, CCND2, VEGFA, ZEB2, ST8SIA4;	Proliferation, migration, invasion, apoptosis, cell cycle, EMT	Tissues: 54 NT Cells: ACHN, 786-O, 769-P, A498, Caki-1, HK-2	[100–104]
SBF2-AS1	miR-338-3p	ETS1	Proliferation, migration, invasion, apoptosis, autophagy	Tissues: 46 NT Cells: Caki-1, UT14, UT16, UT33a, 768-O, HK-2	[105]
MIAT	miR-29c	LOXL2	Proliferation, migration, invasion	Tissues: 45 NT Cells: Caki-1, ACHN, 786-O, HK-2	[106]
ARAP1-AS1	miR-361-3p	PGF	Proliferation, migration, apoptosis	Tissues: 16 NT Cells: HK-2, Caki-1, A498	[107]
MIR155HG	miR-155-5p, miR-155-3p	MMP2, MMP9	Proliferation, migration, invasion	Cells: HKC-5, LoMet-ccRCC, 786-O, A498, Caki-1, HK-2	[108]
DUXAP8	miR-126	CED-9	Proliferation, invasion	Cells: A498, 786-O	[109]
SNHG16	miR-1303-p	STARD9	Proliferation, apoptosis	Tissues: 45 NT Cells: A498, 786-O, Caki-1, OSRC-2, HK-2	[110]
DLX6-AS1	miR-26a	PTEN	Proliferation, apoptosis	Tissues: 52 NT Cells: A498, ACHN, Caki-1, Caki-2, 786-O, G401, HK-2	[111]
EMBP1	miR-9-5p	KLF4, NANOG, CCNE2	Proliferation, migration, invasion, EMT	Tissues: 65 NT Cells: ACHN, Caki-1, HK-2	[112]
LINC02738	miR-20b	SOX4	Proliferation, invasion, apoptosis	Tissues: 50 NT Cells: ACHN, 786-O, OS-RC-2, Caki-2, HK-2	[113]
GAPLINC	miR-135b-5p	CSF1	Proliferation, migration	Cells: A498, OSRC-2, ACHN, 786-O, Caki-1, HK-2	[114]
SNHG4	miR-204-5p	RUNX2	Proliferation, migration, invasion, apoptosis	Tissues: 99 NT Cells: Caki-1, Caki-2, ACHN, 786-O, 769-P, HK-2	[115]
TTN-AS1	miR-195	Cyclin D1	Proliferation, cell cycle	Tissues: 145 NT Cells: ACHN, 786-O, SN12-PM6, HK-2	[116]
ZFPM2-AS1	miR-130a-3p	ESCO2	Proliferation, migration, invasion, apoptosis	Tissues: 60 NT Cells: 786-O, KETR3, G401, HK-2	[117]
SLC16A1-AS1	miR-143-3p	SLC7A11	Proliferation, migration, invasion	Cells: 786-O, A498, Caki-1, HK-2	[118]
DLEU2	miR-30a-5p	ZEB2	Proliferation, invasion	Tissues: 40 NT Cells: ACHN, Caki-1, 769-P, 786-O, HK-2	[33]
LINC01232	miR-204-5p	RAB22A	Proliferation, migration, invasion	Tissues: 122 NT Cells: Caki-1, A498, KN-41, 786-O, ClearCa-1, HK-2	[119]
GIHCG	miR-499a-5p	XIAP	Proliferation, migration, apoptosis, cell cycle	Cells: HK-2, Caki-1, 786-O, A498, SN12C-PM6	[40]
CYTOR	miR-136-5p	MAT2B	Proliferation, invasion, apoptosis	Cells: 786-O, Caki-1	[120]
CASC19	miR-532	ETS1	Proliferation, migration, apoptosis	Tissues: 51 NT Cells: Caki-1, A498,786-O	[121]
FTX	miR-4429	UBE2C	Proliferation, migration, invasion, cell cycle	Tissues: 51 NT Cells: A498, A704, SN12C, 769-P	[42]

^a^Epithelial-Mesenchymal Transition

^b^Paired tumor and adjacent tissues

### Role in inhibiting tumor progression

Several studies have revealed that lncRNAs severed as a suppressive factor for the development of ccRCC (Fig. 2). Ye et al. reported that lncRNA LET was downregulated in ccRCC tissues and cells, which mediated the tumor suppression by directly binding with miR-373-3p to reduce the levels of dickkopf-1 (DKK1) and tissue inhibitor of metalloproteinase-2 (TIMP2) [122]. Overexpression of LET could repress cell cycle and induce apoptosis of ccRCC cells in vitro, and inhibit ccRCC tumor growth in vivo [122]. DKK1 is a member of the dickkopf family, which can inhibit Wnt signaling and regulate immune cells in the tumor microenvironment [123]. Wnt signal pathway plays a crucial role in embryonic development and regulates nephrogenesis in mesenchymal cells [124]. TIMP2 is a member of the TIMP family, which is a natural inhibitor of the matrix metalloproteinases and directly suppresses tumor metastasis by degrading extracellular matrix and basal membrane [125]. Moreover, TIMP2 is downregulated in ccRCC and inhibits the proliferation, migration and invasion of ccRCC cells [126].

LncRNA TCL6 level has been found to be decreased in ccRCC tissues, which inhibited ccRCC metastasis via interacting with miR-155 to influence Src/Akt pathway [12]. The Src/Akt pathway participates in the carcinogenesis and development of ccRCC, whose activation contributes to the malignant phenotypes and tumor progression of ccRCC [127]. Zeng et al. reported that lncRNA 00312 was remarkably downregulated in ccRCC tissues and cell lines compared to the normal controls, which was significantly correlated with the unfavorable prognosis, including tumor size, pathological grade and tumor-node-metastasis (TNM) stage [128]. Additionally, lncRNA 00312 could also act as a sponge of miR-34a-5p to modulate argininosuccinate synthetase 1 (ASS1) expression, thereby promoting cell apoptosis and alleviating the development of ccRCC [128]. LncRNA NBAT1 has been confirmed that it was significantly decreased in ccRCC cells, which could suppress cell proliferation and metastasis through NBAT1/miR-346/glycogen synthase kinase-3β (GSK-3β) axis [11]. Moreover, miR-346 increase or GSK-3β silencing in ccRCC cells could reverse NBAT1-mediated inhibitory effect on cell proliferation, migration, and invasion [11]. The binding of lncRNA GAS5 to miR-21 upregulates the expression of sex-determining region Y-box protein 5 (SOX5), which in turn increases the sorafenib sensitivity of ccRCC [129]. Thus, in the lncRNA/miRNA/mRNA axis, lncRNAs bind to oncogenic miRNAs act as suppressors for ccRCC progression (Table 2).

**Table 2:** Role of lncRNA/miRNA/mRNA axis in inhibiting ccRCC progression

LncRNA	MiRNA	Gene	Biological behavior of ccRCC	Samples	Refs.
LET	miR-373-3p	DKK1, TIMP2	Proliferation, apoptosis, cell cycle	Tissues: 16 NT^a^ Cells: Caki-1, 786-O, 769-P, HEK-293T	[122]
TCL6	miR-155	Src	Proliferation, migration, invasion, apoptosis, cell cycle, EMT^b^	Cells: Caki-1, 786-O, HK-2	[12]
LncRNA 00312	miR-34a-5p	ASS1	Proliferation, invasion, apoptosis	Tissues: 47 NT Cells: A498, ACHN, 786-O, 769-P, HK-2	[128]
NBAT1	miR-346	GSK-3β	Proliferation, migration, invasion	Cells: 786-O, ACHN, Caki-1, Caki-2, HK-2	[11]
PENG	miR-15b	PDZK1	Proliferation	Tissues: 90 NT Cells: ACHN, 769-P, HEK-293	[130]
MEG3	miR-7	RASL11B	Proliferation, migration, invasion, cell cycle, apoptosis	Tissues: 72 NT Cells: A498, 786-O, HK-2	[131]
LINC01939	miR-154	Notch	Proliferation, migration, apoptosis	Tissues: 18 NT Cells: ACHN, Caki-1	[132]
ENTPD3-AS1	miR-155-5p	HIF-1α	Proliferation, migration	Tissues: 105 NT Cells: 786-O, A498, 769-P, ACHN, OS-RC-2	[133]
XIST	miR-106b-5p	P21	Proliferation, cell cycle	Tissues: 50 NT Cells: ACHN, Caki-1, Caki-2, 786-O, HK-2	[134]
ASB16-AS1	miR-185-5p, miR-214-3p	LARP1	Proliferation, migration, invasion	Tissues: 42 NT Cells: A498, 786-O, 769-P, Caki-1, OS-RC-2, ACHN, HEK-293T, HK-2	[135]
COL18A1-AS1	miR-1286	KLF12	Proliferation, migration, invasion	Tissues: 50 NT Cells: ACHN, A498, Caki-1, OS-RC-2, 786-O, HK-2	[136]

^a^Paired tumor and adjacent tissues

^b^Epithelial-Mesenchymal Transition

### Clinical significance of lncRNAs and miRNAs

To date, effective biomarkers for the early diagnosis and prognosis of ccRCC are still scarce in the clinic. Biomarkers, especially in the serum, have attracted great attention for predicting the initiation and development of ccRCC, because they are easy to obtain and assess. Therefore, identification of valuable biomarkers may resolve the difficulties in diagnosis and therapy for ccRCC.

Recent studies have indicated that lncRNAs are potential diagnostic and prognostic biomarkers for ccRCC (Table 3). He et al. reported that lncRNA GIHCG was markedly upregulated in the tissues and serums of ccRCC patients when compared to the healthy control group (AUC = 0.886, 95% CI: 0.812–0.959), with a sensitivity and specificity of 80.7% and 84.8%, respectively. Moreover, the high level of GIHCG in serum was positively correlated with the advanced TNM stages (P = 0.028), Fuhrman grades (P = 0.032), and a worse OS (P = 0.038) [137]. A recent study has demonstrated that the combination of five lncRNAs (LET, PVT1, PANDAR, PTENP1 and LINC00963) could discriminate ccRCC patients from normal controls, with a high specificity of 88.9% and a sensitivity of 79.2%; this finding included 71 ccRCC patients, 8 patients with benign renal tumors, and 62 healthy controls [138]. Tissue lncRNA OTUD6B-AS1 was significantly downregulated in ccRCC patients and exhibited obvious discrimination in the diagnosis (AUC = 0.792, 95% CI: 0.715–0.870) with a sensitivity of 77.3% and specificity of 81.4% [139]. The expression of lncRNA CADM1-AS1 was decreased in ccRCC tissues, and its downregulation was positively related to the AJCC stage (P = 0.039) and lower survival rate of ccRCC patients (P < 0.05). Furthermore, a multivariate analysis found that CADM1-AS1 was an independent prognostic factor for OS (HR = 0.211, 95% CI: 0.088–0.504) [140]. In the metastatic ccRCC patients, lncRNA PGM5-AS1 was reduced, and exerted as an independent prognostic predictor for OS (HR = 2.897, 95% CI: 1.275–4.387) and DFS (HR = 2.875, 95% CI: 1.185–4.462) [141]. LncRNA TCL6 levels in tissues were decreased in ccRCC, which were notably associated with the tumor stages, distant and lymphatic metastasis. Additionally, TCL6 is an independent predictor for Fuhrman grade of ccRCC patients (HR = 4.05, 95% CI: 1.08–15.17) [142].

**Table 3:** Potential lncRNAs and miRNAs biomarkers in ccRCC

Samples	NcRNAs		Sensitivity (%)	Specificity (%)	Refs.
Tissue	LncRNA	APOC1P1	73.3	93.3	[149]
		OTUD6B-AS1	77.3	81.4	[139]
		ZNF180-2	90.0	55.9	[150]
		FZD1-2	85.5	94.2	[151]
		BMP2-2	85.5	100.0	[151]
		SPAM1-6	83.6	94.2	[151]
		ITPR2-3	90.9	96.2	[151]
		CPN2-1	90.9	98.1	[151]
		TTC34-3	98.1	96.4	[151]
		ACACA-1	94.2	100.0	[151]
		LCP2-2	98.1	89.1	[151]
		FOXG1-2	96.2	89.1	[151]
		RP3-368B9.1.1-1	86.5	94.5	[151]
		SLC30A4-1	90.9	96.2	[151]
		Fer1L4	73.2	95.5	[152]
	miRNA	Combination of miR-224-5p, miR-34b-3p and miR-182-5p	80.3	66.3	[153]
		miRNA-135a-5p	45.5	81.1	[154]
		miR-720	80.0	100.0	[155]
		miR-129-3p	75.9	62.1	[156]
		miR-182-5p	90.0	97.0	[81]
		Combination of miR-21 and miR-194	80.0	97.5	[157]
		miR-142-3p	62.0	56.0	[158]
		miR-21	90.0	83.0	[147]
		Combination of miR-10b, miR-139-5p, miR-130b and miR-199b	76.0	100.0	[159]
Serum	LncRNA	LET	70.8	59.3	[138]
		LINC00887	71.1	89.9	[160]
		PVT1	70.8	63.0	[138]
		PANDAR	75.0	63.0	[138]
		PTENP1	79.2	77.8	[138]
		Linc00963	83.3	66.7	[138]
		GIHCG	87.0	84.8	[137]
	miRNA	Combination of miR-378 and miR-210	80.0–83.8	57.1–78.0	[145, 161]
		Combination of miR-10a-5p, miR-10b-5p and miR-223-3p	86.7	75.0	[162]
		Combination of miR-21-5p, miR-150-5p, miR-145-5p and miR-146a-5p	90.8	93.8	[163]
		Combination of miR-378 and miR-451	81.0	83.0	[146]
		miR-1233	77.4–81.0	37.6–76.0	[164, 165]
		miR-210	67.5–82.5	62.2–80.0	[144, 165–167]
		miR-106a	78.1	75.0	[168]
		miR-206	83.8	57.1	[161]
		miR-34a	80.8	80.0	[148]
		miR-141	75.0	73.3	[148]
Urine	miRNA	miR-15a	98.1	100.0	[169]
		miR-30a-5p me	63.0	67.0	[170]
		miR-210	57.8	80.0	[171]
		miR-30c-5p	68.6	100.0	[172]
		Combination of miR-122, miR-1271 and miR-15b	100.0	86.0	[173]
		let-7	71.0	81.0	[174]
		Combination of miR-122-5p, miR-1271-5p and miR-15b-5p	96.0	65.0	[175]
Plasma	miRNA	miR-210	60.9	73.1	[176]
		miR-221	71.4	65.0	[176]
		miR-1233	39.1	92.6	[176]
		miR-424-3p	75.0	81.8	[177]
		miR-144-3p	87.1	83.0	[178]

MiRNAs have also been reported as potential biomarkers for diagnosis, prognosis, and therapy of ccRCC (Table 3). A systematic analysis has identified that there were 118 miRNAs that could be diagnostic biomarkers, 28 miRNAs were prognostic biomarkers, and 80 miRNAs were therapeutic biomarkers [143]. Serum levels of miR-210 were particularly elevated in ccRCC patients, which could be a diagnostic biomarker for ccRCC with a sensitivity of 67.5%, and a specificity of 70% [144]. Moreover, a combination of serum miR-210 and miR-378 could definitely enhance the discriminatory ability of ccRCC, with a sensitivity and specificity of 80% and 78%, respectively [145]. The expression level of serum miR-378 was significantly upregulated in ccRCC patients with an AUC of 0.71, and its overexpression was positively correlated with DFS and clinical stage [145, 146]. Moreover, a combination of serum miR-378 and miR-451 increased the diagnostic efficiency (AUC = 0.86), and a sensitivity of 81% and specificity of 83% [146]. Tissue miR-21 levels were associated with the histological classification of RCC, such as, ccRCC, pRCC, chRCC, and eosinophilia, which could be a diagnostic biomarker with a specificity of 90% (95% CI: 0.639–0.981) and sensitivity of 83% (95% CI: 0.535–0.976%) [147]. In addition, miR-34a and miR-141 were obviously decreased in the serum samples of ccRCC patients, which might be a diagnostic biomarker for ccRCC with a sensitivity of 80.76% and 75%, and specificity of 80% and 73.33%, respectively [148]. Hence, lncRNAs combined with miRNAs might be a promising strategy for ccRCC diagnosis and prognosis.

### Role in drug resistance

Radical nephrectomy and nephron-preserving surgery are the best therapeutic strategies for ccRCC patients at the early stage; however, approximately 30% of patients were initially diagnosed at the metastatic stage [4]. In that case, targeted drugs such as sunitinib, sorafenib, everolimus, are often recommended for their treatments [179]. Nevertheless, a large number of ccRCC patients are exclusively resistant to the targeted therapy, which leads to therapeutic failure and little clinical benefit. Although systemic treatment has been implemented in ccRCC patients, drug resistance is still the primary problem that needs to be overcome [180]. Many studies have proposed that lncRNA/miRNA/mRNA axis has a pivotal role in drug resistance of ccRCC (Table 4).

**Table 4:** LncRNA/miRNA/mRNA axis in ccRCC drug resistance

LncRNA	Therapy	Mechanism	Resistance	Refs.
LINC00461	Sunitinib	miR-942/SALL1, METAP1, DCAF11	Enhance	[181]
SARCC	Sunitinib	miR-143-3p/AKT, MMP13, K-RAS, p-ERK	Weaken	[182]
ARSR	Sunitinib	miR-34/AXL, miR-449/c-MET	Enhance	[183]
HOTAIR	Sunitinib	miR-17-5p/Beclin1	Enhance	[184]
ADAMTS9-AS2	Cisplatin and 5-Fluorouracil	miR-27a-3p/FOXO1	Weaken	[21]
MALAT1	Sunitinib	miR-362-3p/G3BP1	Enhance	[37]
LINC02532	Radiotherapy	miR-654-5p/YY1	Enhance	[185]
GAS5	Sorafenib	miR-21/Sox5	Weaken	[129]
KIF9-AS1	Sorafenib	miR-497-5p/autophagy signaling	Enhance	[22]
PLK1S1	Sorafenib	miR-653/CXCR5	Enhance	[186]
NEAT1	Sorafenib	miR-34a/c-Met	Enhance	[187]

LncRNA ARSR was upregulated in ccRCC cells and its high expression enhanced sunitinib resistance [183]. In this process, lncARSR functions as a ceRNA and sponges miR-34 and miR-449, then induces the expression of AXL and c-MET. LncRNA HOTAIR expression was markedly elevated in ccRCC sunitinib-resistant cells and increased Beclin1 expression by competing with miR-17-5p, thus enhancing sunitinib resistance [184]. Moreover, lncRNA MALAT1/miR-362-3p and LINC00461/miR-942 axis have also been shown to enhance sunitinib resistance. The levels of lncRNA SARCC were increased in sunitinib-treated ccRCC cells, and high expression of SARCC enhanced the sensitivity of ccRCC cells to sunitinib [182]. Mechanistically, SARCC interacted with androgen receptor (AR) and diminished its stability, thereby inhibiting miR-143-3p transcription and downstream signal activation [182]. Additionally, SARCC also regulates AR/HIF-2α/c-Myc axis to promote cell proliferation and ccRCC progression [188]. ccRCC cells with high lncRNA KIF9-AS1 expression enhanced sorafenib resistance by sponging miR-497-5p and activating transforming growth factor-β (TGF-β) signaling [22]. LncRNA GAS5 was downregulated in sorafenib-resistant ccRCC cells, which could upregulate the expression of SOX5 by binding with miR-21 and weaken the sorafenib sensitivity [129]. SOX5 belongs to the SOX family, which contains more than 20 members and some are highly expressed in chemotherapy-resistant cells [189]. Knockdown of lncRNA LINC02532 increases radiosensitivity in ccRCC cells by regulating the miR-654-5p/Yin Yang-1(YY1) axis [185]. YY1 is a DNA/RNA binding transcription factor, and plays an important role in tumorigenesis and radiation resistance [190]. Overexpression of lncRNA ADAMTS9-AS2 in ccRCC cells reduced 5-fluorouracil resistance via sponging miR-27a-3p to upregulate forkhead box protein O1 (FOXO1) [21]. FOXO1 is a transcription factor for tumor suppressor, which is inactivated in tumor cells and acts as a target of the AR/ERβ pathway [191]. In ccRCC cells and tissues, the FOXO1 level was decreased, and overexpression of FOXO1 could induce cell cycle arrest and promote apoptosis. Additionally, FOXO1 expression is closely associated with the pathological classification, tumor grade and tumor stage of ccRCC [192].

### LncRNA/miRNA/mRNA axis in the tumor microenvironment of ccRCC

**Fig. 3:**
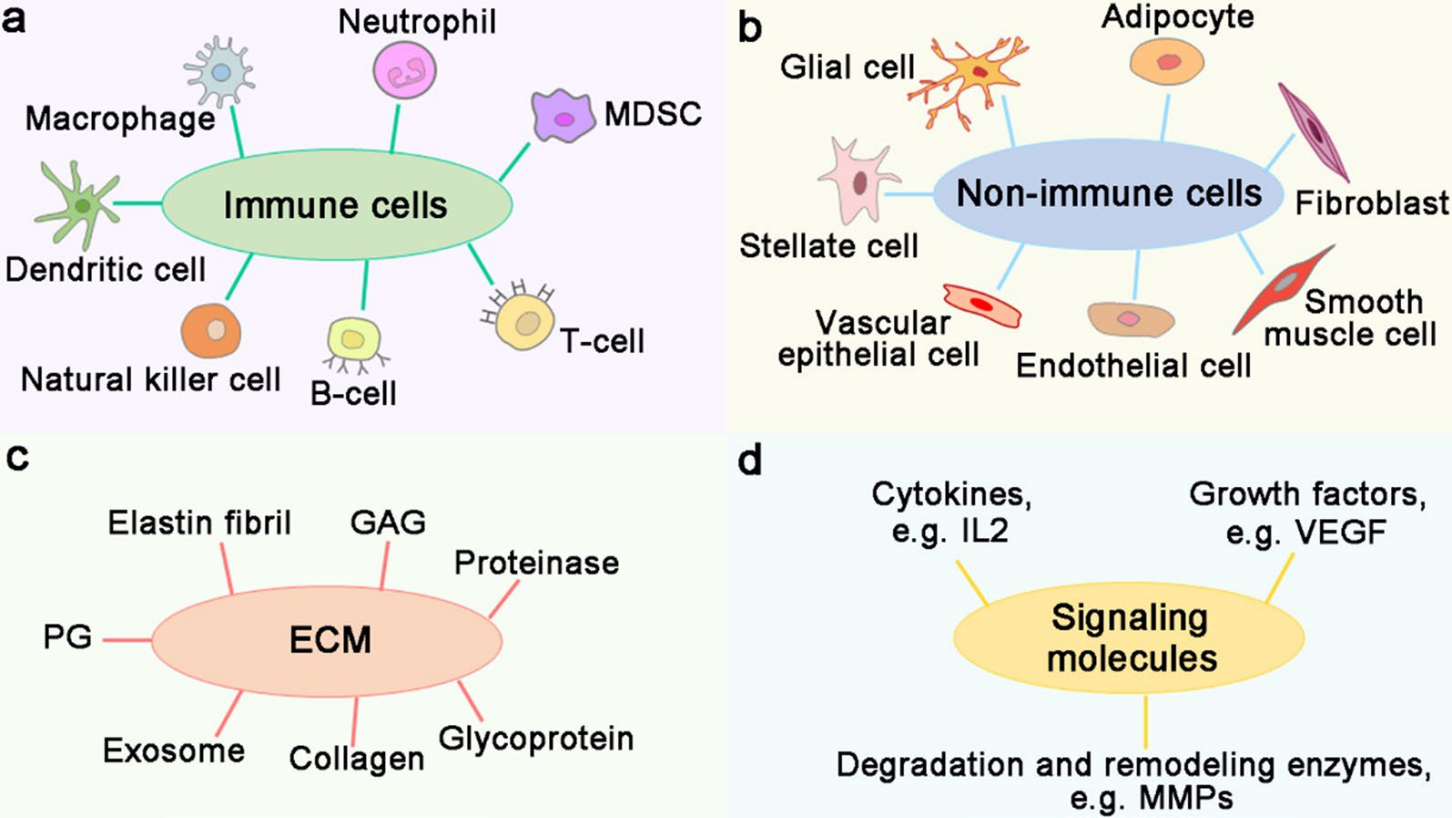
Schematic diagram of TME components. TME consists of immune cells, non-immune cells, extracellular matrix (ECM) and signaling molecules. **a** Immune cells in the TME are composed of macrophages, neutrophils, myeloid-derived suppressor cells (MDSCs), T-cells, B-cells, natural killer cells and dendritic cells. **b** Non-immune cells in the TME mainly include vascular epithelial cells, endothelial cells, fibroblasts, adipocytes, glial cells, stellate cells and smooth muscle cells. **c** ECM refers to the microenvironment supporting the survival and growth of tumor cells, which consists of collagen, elastin fibrils, proteinases, proteoglycans (PGs), glycoproteins, glycosaminoglycans (GAGs) and exosomes. **d** Signaling molecules in the TME include cytokines (e.g. IL2), growth factors (e.g. VEGF) and degradation and remodeling enzymes (e.g. MMPs)

**Fig. 4:**
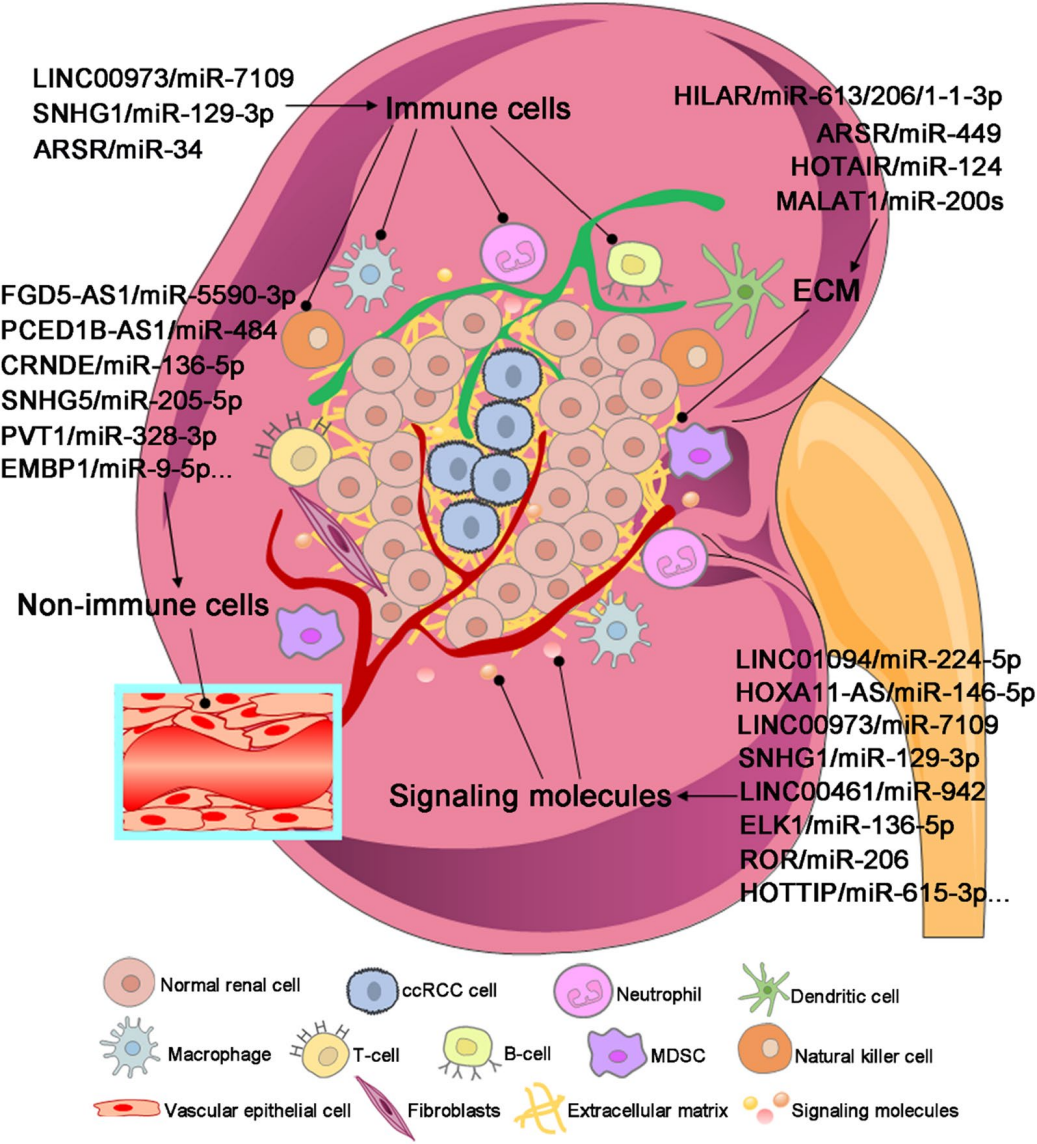
LncRNA/miRNA/mRNA axis in the TME of ccRCC. LncRNA/miRNA/mRNA axis plays a vital role in regulating the TME of ccRCC, such as immune cells, non-immune cells, ECM, and signaling molecules; therefore, the alteration of this network has a significant effect on the initiation and progression of ccRCC

To date, it is still difficult to eradicate the malignant tumors because they are located in the dynamic tumor microenvironment (TME), which can sustain the nutrient demand of tumor cells and help them escape from the directly killing of immune cells. Hence, TME is vital for tumor formation, growth, angiogenesis, metastasis, and influences therapeutic efficacy of drugs [193]. There are many components in the TME, which can be divided into immune cells, non-immune cells, extracellular matrix (ECM) and signaling molecules (Fig. 3). As tumor cells have close communication with the surrounding TME, targeting the components in the TME is currently a research interest for tumor therapies. In recent years, numerous studies have revealed that lncRNA and miRNA network performs an important role in modulating the TME of ccRCC (Fig. 4).

### As a modulator of immune cells

Tumor immunotherapies aim to increase the responses of immune cells, which have already become a hotspot for recent studies. Immune escape is helpful for the growth and metastasis of tumor cells, and decreases the responses to immunotherapy, eventually leading to the poor prognosis of patients [194]. Immune cells in the TME consist of macrophages, neutrophils, dendritic cells, B cells, T cells and other immune cells [195]. Many studies have confirmed that immune cells are pivotal in the progression of ccRCC (Fig. 4) [59, 97, 100]. Siglec-15 is an immunosuppressive molecule, which inhibits antigen-specific T cell reactions in vivo and in vitro, and plays an important role in the TME [196]. One study reported that lncRNA LINC00973 was upregulated in Siglec-15 positive ccRCC, which could induce the expression of Siglec-15 by sponging miR-7109, then enhance the tumor immune suppression. Moreover, overexpression of Siglec-15 in ccRCC cells could significantly inhibit the secretion of IL-2 by Jurkat cells [59]. IL-2 deficiency led to a defect in the homeostasis of CD25^+^ CD4^+^ regulatory T cells and T lymphocytes, which was closely associated with the initiation of autoimmune diseases [197]. Tian et al. have identified that lncRNA SNHG1 was highly expressed in ccRCC, which could enhance the immune escape of ccRCC cells via binding with miR-129‐3p increase the expression of STAT3 [97]. On the other hand, silencing of SNHG1 could increase the secretion of immune-related factors, such as IFN-γ, TNF-α and IL-2. Moreover, knockdown of SNHG1 in ccRCC mice could substantially increase the infiltration of CD8^+^ T cells, and prolong the overall survival of ccRCC mice [97]. Similarly, lncARSR was upregulated in ccRCC and could promote M2 polarization of macrophages by activating the STAT3 signal pathway. Its high expression in ccRCC mice could elevate the levels of CD206 and Ki67 [198]. Additionally, lncARSR acts as a ceRNA of miR34/miR449, which could upregulate the expression of AXL and c-MET to facilitate the progression of ccRCC [183].

### As a modulator of non-immune cells

Epithelial cells are one subtype of the non-immune cells in the TME, which are the most important components in the EMT process for tumor metastasis. In the EMT process, epithelial cells acquire the mesenchymal and fiber-like characteristics, then decrease their intracellular adhesion and increase their metastasis and invasion abilities, eventually transforming into mesenchymal cells to further facilitate tumor metastasis [199]. Studies have shown that lncRNA/miRNA/mRNA axis can influence the EMT process by regulating the epithelial cells (Fig. 4) [55, 62, 64]. Yang et al. have revealed that lncRNA FGD5-AS1 was upregulated in metastatic ccRCC patients. High expression of FGD5-AS1 could promote the migration, invasion and EMT process of ccRCC cells by absorbing miR-5590-3p, thereby activating the ERK/AKT signaling pathway. Otherwise, silencing of FGD5-AS1 or miR-5520-3p could lead to the increase of E-cadherin and decrease of Vimentin [64]. A recent study reported that lncRNA CDKN2B-AS1/miR-141 axis could inhibit the migration, invasion and EMT process of ccRCC cells via reducing the expression of Cyclin D1 and Cyclin D2 [62]. Importantly, the downregulation of Cyclin D could promote the protein degradation of Ras-related C3 botulinum toxin substrate 1 (RAC1) and p-Paxillin. RAC1 is a member of the Rho family of small G proteins, and its activation can influence many cellular processes, such as cytoskeleton reconstruction, cell adhesion and cell apoptosis [200]. Moreover, RAC1 is closely correlated with tumor differentiation, stage and metastasis of ccRCC [201]. Paxillin is a multifunctional cytoskeletal protein that is involved in cell adhesion and highly phosphorylated in the tumor tissues and cells. It has been demonstrated that Paxillin plays an important role in tumor metastasis, which can recruit signaling molecules involved in cell movement and adhesion [202]. Additionally, lncRNA CRNDE expression was considerably elevated in the ccRCC tissues than that in the normal controls, which could promote tumor metastasis by sponging miR-136-5p to activate the EMT process [55].

### As a modulator of ECM

ECM functions as a crucial factor in the crosstalk between tumor and TME. It is a complex system that is composed of many components, including exosomes, collagen, proteinases, glycoproteins, elastin fibrils, glycosaminoglycans and proteoglycans [203]. Among the components in ECM, exosome is a direct signal transmission between tumor cells and TME, which is beneficial for tumor growth and metastasis. Studies have shown that tumor cells can coordinate different cell behaviors to escape the killing by secreting exosomes to ECM (Fig. 4) [44, 198, 204–206]. These secreted exosomes contain numerous information, such as nucleic acids (lncRNAs, miRNAs), growth factors, proteins and lipids, which can be transferred from the donor to the recipient cells [207]. Recently, many studies have focused on the identification of the differential lncRNAs and miRNAs in the exosomes, which may be potential important substances for understanding the mechanism of tumor immune escape.

Jagged1, a ligand of Notch, which can influence cell apoptosis through regulating the Notch signaling pathway [208]. LncHILAR was down-regulated in ccRCC and transmitted by exosomes, whose function was a ceRNA for miR-613, miR-206 and miR-1-1-3p to enhance the expression of Jagged1. Under the hypoxic condition, lncHILAR transferred by exosomes could significantly enhance the invasive abilities of ccRCC cells [44]. LncARSR was highly expressed in ccRCC-derived exosomes, which contributes to the secretion of cytokines, macrophage phagocytosis and angiogenesis [198]. Moreover, lncARSR acted as a ceRNA of miR34/miR449, leading to the elevated expression of AXL and c-MET [183]. Jin et al. have found that lncRNA MALAT1 in exosomes was an important medium for the communication between ccRCC cells and ECM. Exosomes carrying MALAT1 could accelerate the cell viability, migration, invasion and EMT process by inhibiting the activation of transcription factor ETS1 and TFCP2L1 [204]. Additionally, MALAT1 also exerted as a ceRNA by sponging miR-200 s, miR-194-5p and miR-362-3p to induce the expression of ZEB2, ACVR2B and G3BP1 [10, 36, 37]. LncRNA HOTAIR could be released from the tumor cells via exosomes, then transmitted to the endothelial cells to promote tumor angiogenesis [205]. In ccRCC cells, HOTAIR could upregulate the expression of ST8 alpha-N-acetyl-neuraminidase alpha-2,8-sialyltransferase 4 (ST8SIA4) by binding with miR-124 [103]. ST8SIA4 is a member of glycosyltransferase family 29, which is involved in the synthesis of polysialic acid, a modulator of the adhesive properties of neural cell adhesion molecule [209]. In ccRCC cell lines and tissues, ST8SIA4 was significantly upregulated, and its high expression could promote the proliferation and metastasis of ccRCC cells in vitro and in vivo [103, 210]. A recent study has reported that lncRNA IGFL2-AS1 can be packaged into extracellular vesicles (EVs), thereby transmitting the information of sunitinib resistance to other sunitinib-sensitive RCC cells [206]. Meanwhile, IGFL2-AS1 also exerted its function as a tumor promoter by regulating miR-802/cAMP-regulated phosphoprotein 19 (ARPP19) axis [211].

### As a modulator of signaling molecules: growth factors

Growth factors are another subtype of transmitters in the crosstalk between tumor cells and TME. Tumor cells maintain their growth by absorbing the essential nutrition from the surrounding blood vessels; thus, angiogenesis is necessary for the initiation and progression of different tumors [212]. Angiogenic growth factors, such as VEGF, PDGF and fibroblast growth factor 2 (FGF2), interact with their receptors to activate the downstream signaling pathways to regulate angiogenesis [8]. It has been reported that lncRNAs and miRNAs have an interaction with multiple angiogenic growth factors, which can influence ccRCC angiogenesis by regulating their expressions (Fig. 4) [45, 77, 85, 94, 107]. VEGF was overexpressed in ccRCC, which could stimulate the formation of blood vessels through the VHL-HIF pathway [213]. The high level of VEGF in the serum was negatively correlated with the tumor stage, pathological grade and overall survival of ccRCC patients [214]. LncRNA ROR was significantly up-regulated in ccRCC cells and tissues, which could promote cell proliferation, migration and angiogenesis by regulating miR-206/VEGF axis, thereby facilitating the progression of ccRCC [85]. LncRNA TUG1 was highly increased in ccRCC and acted as a ceRNA sponging miR-299-3p to induce the expression of VEGFA to promote tumor angiogenesis. Knockdown of TUG1 could inhibit the proliferation, migration and angiogenesis of ccRCC cells in vitro, and similar results were obtained in ccRCC xenograft mice [45]. Placental growth factor (PGF) is a member of the VEGF subfamily, which cooperates with VEGF to promote the formation of blood vessels [215]. PGF amount was greatly increased in the serum samples of ccRCC patients, and its high level was notably related to the poor prognosis of ccRCC [216]. LncRNA ARAP1-AS1 could induce the expression of PGF by sponging miR-361-3p, and promote the proliferation, wound healing and invasion of ccRCC cells [107]. FGF2 is a hematopoietic growth factor that positively regulates the hematopoietic function of various cells, including stromal cells, hematopoietic progenitor cells and blood cells [217]. LncRNA PCGEM1 level was elevated in ccRCC cells, which could promote the proliferation and metastasis of ccRCC cells by binding with miR-433-3p to upregulate FGF2 [77]. Insulin-like growth factor 2 (IGF-2) participates in the regulation of TME by enhancing the anti-inflammatory properties of macrophages. Also, IGF-2 regulates the angiogenesis of vascular endothelial cells, leading to the activation of fibroblasts [218]. LncRNA HOTTIP functioned as a ceRNA for miR-615-3p, which could boost the expression of IGF-2. Moreover, HOTTIP was significantly up-regulated in ccRCC, and its elevated expression is negatively correlated with the OS and DFS of ccRCC patients [94].

### As a modulator of signaling molecules: degradation and remodeling enzyme

Matrix metalloproteinases (MMPs) is a large family of zinc-binding proteins, which has been discovered by at least 26 members of this family. Members in the MMP family have the ability to degrade the extracellular matrix and basal membranes; therefore, the alteration of MMPs expression is closely associated with cell differentiation, angiogenesis, extracellular matrix remodeling and metastasis. Studies have found that MMPs can be regulated by various factors, such as hormones, growth factors, cytokines [219]. Currently, lncRNAs and miRNAs also have been demonstrated that they can regulate the expression of many MMP members, then influence the progression of different tumors (Fig. 4) [56, 61, 181]. For example, lncRNA HOXA11-AS could regulate MMP16 by sponging miR-146b-5p, which could promote the proliferation and invasion of ccRCC cells [61]. MMP16 is a membrane protein that is localized in fibroblasts, which is responsible for the degradation of various ECM components and acceleration of the EMT process [220]. Moreover, HOXA11-AS was upregulated in ccRCC tissues and cells, and its high expression was positively correlated with the clinical stage, tumor stage and lymphatic metastasis of ccRCC [61]. MMP2 and MMP7 were found to be increased in ccRCC, and were positively associated with tumor metastasis, pathological grade and clinical stage [221]. Jiang et al. have found that silencing of LINC01094 in ccRCC cells could inhibit the expression of MMP2 and MMP7, then prevent the EMT process [56]. Additionally, LINC01094 acted as a ceRNA of miR-224-5p to induce the expression of chondroitin sulfate synthase 1 (CHSY1), which could enhance the malignant behavior of ccRCC cells. CHSY1 exerts the antagonistic ability for cell apoptosis, and promotes tumor progression by regulating the NF-κB or caspase-3/7 signaling pathway [222]. MMP9 is an important component for the remodeling of extracellular matrix, which plays a critical role in tumor metastasis and progression [223]. It has been reported that MMP9 has an impact on the biological functions of monocytes and related differential cells, and its expression was elevated in ccRCC patients with high abundance of monocytes [224]. LINC00461 was highly expressed in ccRCC cells, and acted as a ceRNA for miRNA-942 to influence the survival of ccRCC patients [181]. Notably, overexpression of miR-942 in metastatic ccRCC cells could promote the secretion of MMP9 and VEGF, which could enhance sunitinib resistance of endothelial cells [225].

## Conclusions

Extensive evidence has revealed that lncRNAs and miRNAs are involved in the diagnosis, prognosis, and drug therapy of ccRCC. Understanding the interaction between lncRNA and miRNA network and TME enables us to deeply understand the initiation, development and drug resistance of ccRCC. Nevertheless, the role of lncRNA/miRNA/mRNA axis in the TME of ccRCC remains poorly understood. Hence, in this review, we mainly focus on the biological function of lncRNA/miRNA/mRNA axis in the progression of ccRCC, then discuss how lncRNA/miRNA/mRNA axis regulate the immune cells, non-immune cells, ECM, growth factors, degradation and remodeling enzymes in TME, and finally highlight their potential application as novel biomarkers and therapeutic targets for ccRCC.

MiRNA can bind to the 3’UTR of target mRNAs, while lncRNA act as a sponge of miRNA to prevent the binding between miRNA and mRNA, which can be defined as lncRNA/miRNA/mRNA regulatory network. LncRNA/miRNA/mRNA axis can regulate the gene expression and signal transduction, and play a critical role in transmitting the information between tumor cells and the surrounding TME, thereby influencing the progression of ccRCC. During the initiation and development of ccRCC, lncRNAs have a bidirectional regulatory function of tumor cells, which combine with oncogenic miRNAs will result in tumor inhibition, and interact with suppressive miRNAs will promote tumor progression. Since lncRNAs and miRNAs are stable in the serum, urine, tissues, and they can directly reflect the characteristics of tumor cells; therefore, lncRNAs and miRNAs can become diagnostic and predictive biomarkers of ccRCC. More importantly, lncRNA/miRNA/mRNA axis has a close interaction with the cellular and non-cellular components in the TME of ccRCC, which can influence the cell proliferation, apoptosis, migration, invasion, angiogenesis, EMT process, and immune responses. The interaction between the components in TME and lncRNA/miRNA/mRNA axis will significantly influence the survival, prognosis and drug sensitivity of ccRCC patients.

To date, most studies remain focus on the role of lncRNA/miRNA/mRNA axis in promoting or inhibiting the progression of ccRCC, and little evidence regarding the lncRNA/miRNA/mRNA axis in regulating the crosstalk between ccRCC cells and TME. Moreover, little information has been presented for the clinical application of lncRNA/miRNA/mRNA axis in ccRCC. Therefore, it is necessary to clarify the clinical significance of the lncRNA/miRNA/mRNA axis in the future. First, the delivery of lncRNAs or miRNAs to ccRCC cells needs to be explored to improve the efficacy of drug therapy. Second, the regulation of lncRNAs or miRNAs to overcome the immune escape in ccRCC cells. Third, the evaluation of the doses and pharmacokinetics of lncRNA-based therapies. In conclusion, further studies of lncRNA/miRNA/mRNA axis will provide more information and novel insights to understand the pathogenesis of ccRCC, which will help us better predict the diagnosis and prognosis of ccRCC.

## Data Availability

Not applicable.

## Abbreviations

RCC: Renal cell carcinoma
ccRCC: Clear renal cell carcinoma
lncRNA: Long non-coding RNA
miRNA: MicroRNA
mRNA: Messenger RNA
pRCC: Papillary RCC
chRCC: Chromophobe RCC
TME: Tumor microenvironment
VHL: Von Hippel-Lindau
HIF: Hypoxia-inducible factor
VEGF: Vascular endothelial growth factor
PDGF: Platelet-derived growth factor
mTOR: Mammalian target of rapamycin
VEGFR: Vascular endothelial growth factor receptor
PDGFR: Platelet-derived growth factor receptor
ncRNA: Non-coding RNA
sncRNA: Small non-coding RNA
3ʹUTR: 3ʹUntranslated regions
FAM193B: Family with sequence similarity 193 member B
ERK: Extracellular signal-regulated kinase
OS: Overall survival
ceRNA: Competing endogenous RNA
TBX15: T-box transcription factor 15
DFS: Disease-free survival
ZEB2: Zinc finger E-box binding homeobox 2
DNA: Deoxyribo nucleic acid
ACVR2B: Activin receptor type 2B
G3BP1: Ras-GTPase-activating SH3-domain-binding protein 1
DARS: Aspartyl-tRNA synthetase
XIAP: X-linked inhibitor of apoptosis protein
DKK1: Dickkopf-1
TIMP2: Tissue inhibitor of metalloproteinase-2
TNM: Tumor-node-metastasis
ASS1: Argininosuccinate synthetase 1
GSK-3β: Glycogen synthase kinase-3β
AUC: ROC curve
AR: Androgen receptor
HIF-2α: Hypoxia-inducible factor-2α
TGF-β: Transforming growth factor-beta
YY1: Yin Yang-1
FOXO1: Forkhead box protein O1
ECM: Extracellular matrix
IFN-γ: Interferon-γ
TNF-α: Tumor necrosis factor-α
IL-2: Interleukin-2
EMT: Epithelial mesenchymal transition
RAC1: Rac family small GTPase 1
EV: Extracellular vesicle
TFCP2L1: Transcription factor CP2-like 1
ST8SIA4: α-2,8-Sialyltransferase 4
FGF2: Fibroblast growth factor 2
PGF: Placental growth factor
IGF-2: Insulin-like growth factor 2
CHSY1: Chondroitin sulfate synthase 1
ADAMTS9-AS2: ADAMTS9 antisense RNA 2
AJCC: American Joint Committee on Cancer
AKT: Protein kinase B
ARAP1-AS1: ARAP1 antisense RNA 1
CADM1-AS1: CADM1 antisense RNA 1
CDKN2B-AS1: CDKN2B antisense RNA 1
c-MET: C-mesenchymal-epithelial transition factor
CRNDE: Colorectal neoplasia differentially expressed
DARS-AS1: Aspartyl-tRNA synthetase antisense RNA 1
FGD5-AS1: FGD5 antisense RNA 1
GAS5: Growth arrest-specific transcript 5
GIHCG: Gradually increased during hepatocarcinogenesis
HIF-1α: Hypoxia inducible factor-1α
HOTAIR: HOX transcript antisense intergenic RNA
HOTTIP: HOXA transcript at the distal tip
HOXA11-AS: HOXA11 antisense RNA
IL-6: Interleukin-6
KIF9-AS1: KIF9 antisense RNA 1
lncARSR: Long noncoding RNA ARSR
lncHILAR: Hypoxia-induced long non-coding RNA associated with RCC
MALAT1: Metastasis-associated lung adenocarcinoma transcript 1
MAPK: Mitogen-activated protein kinase
MMP: Matrix metalloproteinase
NBAT1: Neuroblastoma associated transcript 1
PANDAR: Promoter of CDKN1A antisense DNA damage activated RNA
PCGEM1: Prostate cancer gene expression marker 1
p-ERK: Phosphorylated extracellular signal-regulated kinases
PI3K: Phosphatidylinositol 3-kinase
PTENP1: Phosphatase and tensin homolog pseudogene 1
PVT1: Plasmacytoma variant translocation 1
RNA: Ribonucleic acid
ROR: Regulator of reprogramming
SNHG: Small nucleolar RNA host gene
SARCC: Suppressing androgen receptor in renal cell carcinoma
SOX: Sex-determining region Y-box
SRC: Proto-oncogene tyrosine-protein kinase SRC
STAT3: Signal transducer and activator of transcription 3
TCL6: T-cell leukemia/lymphoma 6
TUG1: Taurine upregulated gene 1
VEGFA: Vascular endothelial growth factor A
UBE2C: Ubiquitin-mediated proteolysis genes
ARPP19: CAMP-regulated phosphoprotein 19
